# The characteristics of nivolumab-induced colitis: an evaluation of three cases and a literature review

**DOI:** 10.1186/s12876-018-0864-1

**Published:** 2018-08-31

**Authors:** Ryosuke Yamauchi, Toshihiro Araki, Keiichi Mitsuyama, Takaaki Tokito, Hidenobu Ishii, Shinichiro Yoshioka, Kotaro Kuwaki, Atsushi Mori, Tetsuhiro Yoshimura, Osamu Tsuruta, Takuji Torimura

**Affiliations:** 1 Division of Gastroenterology, Department of Medicine, Kurume University School of Medicine, 67 Asahi-machi, Kurume, 830-0011 Japan; 2 Inflammatory Bowel Disease Center, Kurume University School of Medicine, 67 Asahi-machi, Kurume, 830-0011 Japan; 3 Division of Respirology, Neurology, and Rheumatology, Department of Internal Medicine, Kurume University School of Medicine, 67 Asahi-machi, Kurume, 830-0011 Japan

**Keywords:** Nivolumab, Immune-checkpoint inhibitor, Diarrhoea, Colitis, Ulcerative colitis

## Abstract

**Background:**

The use of immune-checkpoint inhibitors in cancer treatment has become increasingly common, resulting in an increase in the incidence of related side effects. Diarrhoea and colitis have been previously documented as gastrointestinal tract-related side effects of immune-checkpoint inhibitors. Although PD-1/PD-L1 inhibitors produce fewer side effects than CTLA-4 inhibitors, diarrhoea and colitis continue to be reported. However, little is known about the endoscopic features associated with PD-1/PD-L1 inhibitors. In this report, we describe three cases of colitis induced by a PD-1 inhibitor nivolumab. These cases showed endoscopic findings characteristic of ulcerative colitis (UC). Treatment was in accordance with UC therapy, which resulted in beneficial outcomes.

**Case presentation:**

Three patients with lung cancer treated with nivolumab presented with diarrhoea with (case 2) or without haematochezia (cases 1 and 3). Treatment with nivolumab was ceased and colonoscopy was performed, revealing endoscopic features similar to those of UC. These patients were diagnosed with nivolumab-induced colitis. Case 1 was treated with mesalazine, whereas cases 2 and 3 were treated with corticosteroids. Subsequently, their symptoms improved.

**Conclusions:**

Nivolumab-induced colitis exhibited similar characteristics to UC. Treatment was similar to that for UC and was successful.

## Background

Immune-checkpoint inhibitors, such as anti CTLA-4 antibody, anti PD-1 antibody, and anti PD-L1 antibody, have been shown to extend the survival rate of cancer patients [1–3], and their clinical usage has increased rapidly. These antibodies block the inhibitory signal by binding to the inhibitory receptor or its ligand and enhance the immune response against the tumour. However, blockade of immunity checkpoints is associated with inflammatory side effects known as immune-related adverse events. These events can affect any organ system but typically target the gastrointestinal, hepatic, skin, and endocrine systems [4]. According to previous reports, there is a difference in the frequency of diarrhoea/colitis after blockade between CTLA-4 and PD-1/PD-L1. The incidence of Common Terminology Criteria for Adverse Events (CTCAE) grade 3/4 diarrhoea is 1–2% among patients treated with PD-1/PD-L1 inhibitors compared to 3–6% among patients treated with CTLA-4 inhibitors. Grade 3/4 colitis accounts for 1–3% among patients treated with PD-1/PD-L1 inhibitors compared to 7–9% among patients treated with CTLA-4 inhibitors. These findings suggest that colitis is less frequent during treatment with PD-1/PD-L1 inhibitors than during treatment with CTLA-4 inhibitors [3, 5, 6]. Moreover, little is known about the endoscopic features of PD-1/PD-L1 inhibitors except for what has been documented in the four case reports published to date [7–10]. In this report, we describe three cases of anti-PD-1 antibody nivolumab cessation because of severe colitis and consider the clinical features of this condition.

## Case presentation

As shown in Table 1, all three patients were adult men (case 1: 73, case 2: 78, case 3: 49 years old) with advanced non-small cell lung cancer at our hospital. Nivolumab was administered at a dose of 180 mg every 2 weeks for cases 1 and 3 and 130 mg every 2 weeks for case 2. Symptoms have developed at different times in each case. Case 1 reported grade 3 diarrhoea 15 weeks after the administration. Case 2 reported grade 2 colitis and diarrhoea five times per day for 7 weeks after the administration. Case 3 reported grade 1 diarrhoea after 3 weeks which worsened to grade 2 over time. Symptoms did not improve after nivolumab cessation in these three cases and after administration of probiotics (cases 1 to 3) and antidiarrhoeal drugs (cases 1 and 2). In all cases, infectious diseases were excluded by stool culture.

**Table 1:** Summary of the endoscopic findings from the seven patients diagnosed with nivolumab-induced colitis

	Age/Gender	Tumour Types	Onset^a^	Symptoms	Endoscopic Findings	Disease Location	Histological Findings	Treatment	Outcome
Kubo et al. [7]	82/M	Non-small-cell lung cancer	6 weeks	Diarrhoea and abdominal pain	Reddish and oedematous mucosa with loss of vascularity and ulcerations	Left side of the colon	Inflammatory infiltrates with crypt abscesses and Meissen’s plexus degeneration	Mesalazine	Improved
Takayama et al. [8]	89/M	Melanoma	20 weeks	Diarrhoea	Oedematous mucosa with increased mucous exudate and loss of vascularity	Entire colon	Inflammatory infiltrates with crypt abscesses	Mesalazine PSL^b^	Improved
Takenaka et al. [9]	45/F	Adenocarcinoma of lung	4 weeks	Diarrhoea and abdominal pain	Reddish and oedematous mucosa with ulceration	Left side of the colon	Inflammatory infiltrates with crypt abscesses	PSL Infliximab	Improved
Yanai et al. [10]	51/M	Melanoma	9 weeks	Bloody diarrhoea and abdominal pain	Reddish, oedematous mucosa with increased mucous exudate and loss of vascularity	Entire colon	Inflammatory infiltrates with crypt abscesses and prominent apoptosis	PSL^b^ Infliximab	Improved
Case 1	73/M	Non-small-cell lung cancer	15 weeks	Diarrhoea	Granular mucosa with increased mucous exudate and loss of vascularity	Entire colon	Inflammatory infiltrates with crypt abscesses	Mesalazine	Improved
Case 2	78/M	Adenocarcinoma of lung	7 weeks	Diarrhoea and bleeding	Reddish and oedematous mucosa with loss of vascularity and ulcerations	Left side of the colon	Inflammatory infiltrates with crypt abscesses and cryptitis	PSL^b^	Improved
Case 3	49/M	Adenocarcinoma of lung	3 weeks	Diarrhoea	Reddish, oedematous mucosa with increased mucous exudate and loss of vascularity	Entire colon	Inflammatory infiltrates with epithelial damage	PSL^b^	Improved

^a^ Onset of abdominal symptoms after initiation of treatment with nivolumab

^b^ PSL, prednisolone

**Fig. 1:**
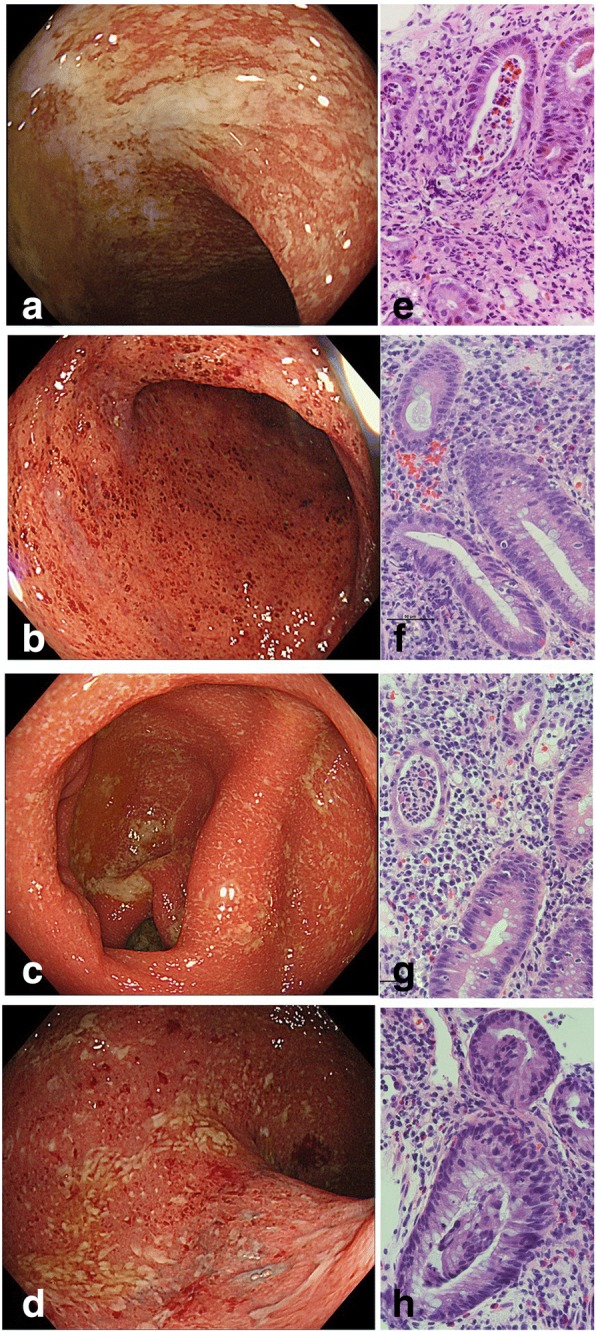
Endoscopic and histopathologic findings. Case 1 (rectum, **a** and **e**), 2 (sigmoid colon, **b** and **f**), 3 (ascending colon, **c** and **g**), and one patient with UC as a comparison (rectum, **d** and **h**). **e**-**h** stained with haematoxylin and eosin. Original magnification of microscopy, × 400

They underwent endoscopy examination. Colonoscopic findings showed persistent inflammation of the entire colon in case 1 (Fig. 1a) and case 3 (Fig. 1c) and left-sided colon in case 2 (Fig. 1b) with a reddish, oedematous mucosa with increased mucous exudate and loss of vascularity (Fig. 1a-c). Histologically, mixed inflammatory infiltrates with crypt abscesses and cryptitis were observed in all cases (Fig. 1e-g). To clarify the similarity between their appearance, representative endoscopic and histological images of ulcerative colitis (UC) are presented in Fig. 1d and h.

As a result, they were diagnosed with nivolumab-induced colitis. Case 1 started treatment with 4000 mg of mesalazine, and then the symptoms and colitis improved. The frequency of diarrhoea then decreased, and endoscopic examination indicated an improvement in the patient’s condition. Cases 2 and 3 were treated with 2 mg/kg/day of methylprednisolone (80 mg/day for case 2, 120 mg/day for case 3). The diarrhoea and colitis gradually improved, prompting us to gradually reduce their corticosteroid treatment.

## Discussion and conclusions

The PD-1 pathway controls autoimmunity and suppresses inflammation. Furthermore, inhibition of the PD-1 pathway in mice leads to various autoimmune diseases [11]. In a phase 1 study of nivolumab use in 296 patients, diarrhoea occurred in 33 patients, including three grade 3/4 events [3]. In another study, the incidence rates of any grade or grade 3/4 diarrhoea in patients who had undergone treatment with nivolumab were 12.7 and 0.5%, respectively. Furthermore, the incidence rates of colitis were 1.0 and 0.7%, respectively [12]. The median time to onset of diarrhoea was 7 weeks in those who were treated with both ipilimumab and nivolumab [13].

The characteristic endoscopic findings of immune-checkpoint inhibitor-induced colitis have been reported previously. Inflammatory changes, such as exudates, granularity, loss of vascular pattern, and ulcerations extending from the rectum to parts or the entire colon, can be observed during colonoscopy in patients treated with ipilimumab [14]. These are similar to findings of inflammatory bowel disease, particularly UC (Fig. 1d). According to recent reports [7–10], similar findings were observed following treatment with nivolumab. We observed the same clinical findings, which included continuous mucosal inflammation, in the patients described in this study (Table 1). As such, the endoscopic images simulate UC to some extent but do not completely resemble it.

Tissue derived from patients with active colitis is histopathologically characterised by marked mixed inflammatory cell infiltrates in the lamina propria, including neutrophils, lymphocytes, plasma cells, and eosinophils [14]. Foci of neutrophilic cryptitis, crypt abscesses, glandular destruction, and erosions of the mucosal surface can be found with occasional ulcers [15, 16]. According to the European Crohn’s and Colitis Organisation guidelines, microscopic diagnosis of UC is based on widespread crypt architectural distortion and a diffuse transmucosal inflammatory infiltrate with basal plasmacytosis, which is eventually associated with an active component and results in cryptitis and crypt abscess [17]. As neither is a characteristic finding, it is difficult to differentiate based on pathological findings. However, Yanai et al. reported that prominent apoptosis in the colorectal epithelium appears to be a characteristic histological finding of nivolumab-induced colitis [10]. To clarify the differences between UC and nivolumab-induced colitis, further evidence derived from reported cases is needed.

As shown in this report, nivolumab-induced colitis was similar to UC in both colonoscopic and histological images. At present, the reason for the similarity is highly speculative since there was no report on the PD-1 pathway in UC. Nancey et al. analysed blood samples from patients for immune-checkpoint inhibitor-induced colitis by flow cytometry, revealing T-cell imbalance including the decrease in regulatory T-cell and the increase in effector T-cells [18]. A similar T-cell imbalance was obtained from UC samples [19]. In PD-1-deficient mice, excessive cytokines are produced from T cells and are reported to cause autoimmune disease onset [20]. Together, excessive cytokine production from activated T cells may partly play an important role in the morphological and pathogenic similarity between the two diseases.

If immune-related colitis is suspected, a gradual approach according to severity is recommended [21]. The National Cancer Institute’s CTCAE has typically been used to define grades of diarrhoea and colitis during clinical treatment. According to previous reports [4, 13, 21, 22], if grade 1 diarrhoea/colitis is diagnosed, immune-checkpoint inhibitors should be administered continuously and symptomatic treatment and careful follow-up should be conducted. If the diarrhoea is of grade 2, where there is increased stool frequency, abdominal pain, mucous faeces, or bloody stool, immune-checkpoint inhibitors should be discontinued and symptomatic treatment should be performed. For grade 2 diarrhoea/colitis, oral administration of systemic corticosteroids (equivalent to 0.5–1 mg/kg/day methylprednisolone equivalent) for > 3 days is recommended. If improvement is observed by systemic steroid administration, the steroid dose is gradually decreased over 4–8 weeks. Despite systemic corticosteroid administration, if improvement is not seen within 3–5 days or if the symptoms worsen, it should be treated as grade 3. For grade 3 diarrhoea, intravenous administration of high-dose systemic steroids (1–2 mg/kg/d methylprednisolone equivalent) is recommended. In refractory cases, immune checkpoint inhibitors cannot be administered [22] and immunosuppressive treatment should be escalated. The potential risk of gastrointestinal perforation should be considered for patients in emergency situations. Infliximab therapy can be considered for patients who do not respond to steroids [10, 23] but cannot be used when gastrointestinal perforation or sepsis is observed.

Interestingly, mesalazine treatment was found to reduce the severity of immune-checkpoint inhibitor-induced colitis symptoms with an improvement in the frequency of diarrhoea and endoscopic findings [7, 8]. The same trend was observed in our patient who was treated with mesalazine, which functions as an inhibitor of inflammatory cytokines as well as prostaglandin-related substances [24, 25]. In this way, mesalazine may improve patient outcomes following nivolumab-induced colitis. Together with the similar inhibitory action of steroids with mesalazine on cytokines [26, 27], the efficacy of mesalazine and steroids on this immune-related colitis may be induced at least in part through the inhibition of inflammatory cytokines.

As described in this series of cases, nivolumab-induced colitis exhibited similar characteristics as UC. Treatment was similar to that for UC and was largely successful.

## Abbreviations

CTCAE: Common terminology criteria for adverse events
UC: Ulcerative colitis
